# CYP2D6 Metabolizer Phenotype Is Associated with Early Antidepressant Discontinuation in the UK Biobank

**DOI:** 10.3390/ph19071028

**Published:** 2026-07-01

**Authors:** Tehila Cohen, Estee Rebibo Demry, Allan H. Young, K. Kleine Schaars, Mario Juruena, Thomas G. Schulze, Jaakko Kaprio, PSY-PGx Consortium, Roos van Westrhenen, Noam Shomron

**Affiliations:** 1 Human Genetics and Computational Medicine, Gray Faculty of Medicine, Tel Aviv University, Tel Aviv 6997801, Israel; tehilaco@tauex.tau.ac.il (T.C.);; 2 Sagol School of Neuroscience, Tel Aviv University, Tel Aviv 6997801, Israel; 3 Institute of Psychiatry, Psychology & Neurosciences, King’s College London, London WC2R 2LS, UK; 4 Division of Psychiatry, Imperial College London, London W12 0NN, UK; 5 Outpatient Clinic Pharmacogenetics, Parnassia Psychiatric Institute, 1062 HN Amsterdam, The Netherlandsr.vanwestrhenen@psyq.nl (R.v.W.); 6 Centre for Human Drug Research, 2333 CL Leiden, The Netherlands; 7 Leiden University Medical Center, 2333 ZA Leiden, The Netherlands; 8 Institute of Psychiatric Phenomics and Genomics (IPPG), LMU University Hospital, LMU Munich, 80336 Munich, Germany; 9 Department of Psychiatry and Behavioral Sciences, Norton College of Medicine, SUNY Upstate Medical University, Syracuse, NY 13210, USA; 10 World Psychiatric Association, 1226 Geneva, Switzerland; 11 Institute for Molecular Medicine Finland (FIMM), Helsinki Institute of Life Science (HiLIFE), University of Helsinki, 00290 Helsinki, Finland; 12 St. John’s National Academy of Health Sciences, Bangalore 560034, India; 13 Institute of Psychiatry, Psychology & Neuroscience (IoPPN), King’s College London, London SE5 8AB, UK; 14 Edmond J Safra Center for Bioinformatics, Gray Faculty of Medicine, Tel Aviv University, Tel Aviv 699780, Israel

**Keywords:** CYP2D6, cytochrome P450, antidepressants, depressive disorder, pharmacogenetics, drug metabolism, treatment discontinuation

## Abstract

**Background/Objectives**: Antidepressant treatment response is highly variable, and CYP2D6 metabolizer phenotype has been proposed as a contributor to this variability. It was examined whether CYP2D6 metabolizer phenotype is associated with real-world antidepressant treatment outcomes in a large population-based cohort. **Methods**: Using genetic and longitudinal primary care prescription data from the UK Biobank, we evaluated associations between CYP2D6 metabolizer phenotype and prescription-based proxies of treatment outcomes, including discontinuation, switching, and side effects. Analyses were stratified by antidepressant and adjusted for demographic covariates. **Results**: Among 26,957 individuals of European ancestry prescribed CYP2D6-metabolized antidepressants, reduced metabolic capacity was significantly associated with early discontinuation of paroxetine (*N* = 5718), venlafaxine (*N* = 2327), and mirtazapine (*N* = 3340). For paroxetine, poor metabolizers had higher odds of discontinuation compared with normal metabolizers and, among discontinuers, were more likely to stop immediately rather than later. Similar early discontinuation signals were observed for venlafaxine, with intermediate metabolizers showing increased risk. Mirtazapine also demonstrated increased odds of early discontinuation among poor metabolizers. No significant association was observed for fluoxetine. Associations with switching were limited, and no significant associations were detected for side effects. **Conclusions**: CYP2D6 variation appears to primarily influence early antidepressant discontinuation within the first 30 days of treatment, particularly for paroxetine, venlafaxine, and mirtazapine, rather than treatment switching or side effects. These findings provide observational support relevant to drug-specific gene interactions and suggest a role for CYP2D6-guided prescribing in clinical practice, notably in the first 30 days of antidepressant treatment.

## 1. Introduction

Major depressive disorder (MDD) is a leading cause of disability worldwide and constitutes a substantial public health burden. Globally, approximately 5.7% of adults experience depression at any given time, with slightly higher estimates of around 6.5% reported in Europe [1,2].

Although antidepressant medications are widely prescribed for major depressive disorder, treatment response is highly variable [3]. Under the current trial-and-error paradigm, many patients experience inadequate symptom improvement despite prolonged treatment trials, while others discontinue therapy early due to adverse effects or perceived lack of benefit [4,5,6,7]. As clinical guidelines recommend waiting at least two to four weeks to determine efficacy, ineffective treatments may be continued for extended periods, contributing to prolonged depressive episodes, patient frustration, and high rates of discontinuation [6]. Identifying factors that contribute to this heterogeneity remains a major challenge in clinical psychiatry.

One proposed contributor to interindividual variability in antidepressant outcomes is genetic variation in drug-metabolizing enzymes. Cytochrome P450 2D6 (CYP2D6) is a key hepatic enzyme involved in the metabolism of several commonly prescribed antidepressants, including selective serotonin reuptake inhibitors and serotonin-noradrenaline reuptake inhibitors such as paroxetine, fluoxetine, mirtazapine, and venlafaxine [8]. Encoded on chromosome 22q13.1, CYP2D6 is responsible for the metabolism of approximately 25% of clinically used drugs despite representing only a minor fraction of total hepatic CYP450 content. The gene is highly polymorphic, with over 100 known allelic variants that result in substantial interindividual variability in enzymatic activity [9,10]. Functional genetic variation in CYP2D6 gives rise to distinct metabolizer phenotypes, ranging from poor to ultrarapid metabolizers, which can substantially influence drug exposure [9]. Reduced or increased metabolic capacity may alter plasma drug concentrations, potentially affecting treatment tolerability, persistence, and clinical response [11].

Pharmacogenetic guidelines provide dosing and prescribing recommendations for CYP2D6-metabolized antidepressants based on predicted metabolizer status [8]. However, despite the availability of such guidelines, evidence linking CYP2D6 metabolizer phenotype to real-world antidepressant treatment outcomes remains mixed, and pharmacogenetic testing and guideline-based dose adjustments are still infrequently implemented in routine clinical practice [12,13]. Prior studies have reported inconsistent associations with clinical response, adverse effects, or treatment changes, often limited by small sample sizes, heterogeneous outcome definitions, or short follow-up periods [10,14,15,16].

Large-scale population cohorts with linked genetic and prescription data offer an opportunity to evaluate the clinical relevance of pharmacogenetic variation under real-world conditions. The UK Biobank provides linked genetic and longitudinal prescription data, enabling investigation of treatment-related outcomes—including discontinuation, switching, and adverse event reporting—as pragmatic proxies of tolerability and persistence in routine clinical practice. A recent UK Biobank analysis demonstrated that pharmacogenetic variation for CYP2C19 can be evaluated using prescription-based proxy outcomes for SSRI treatment response, supporting the utility of this approach in large population datasets [17].

The aim of the present study was to examine the association between CYP2D6 metabolizer phenotype and proxies of antidepressant treatment outcomes in the UK Biobank as part of the Horizon2020 funded PSY-PGx Project [18]. Focusing on antidepressants metabolized, in whole or in part, by CYP2D6, we assessed relationships between metabolizer status and treatment discontinuation, switching, and reported side effects using standardized outcome definitions and guideline-based phenotype classification.

## 2. Results

### 2.1. Study Demographics and CYP2D6 Metabolizer Phenotype Distribution

In total, 26,957 participants had available demographic, clinical and prescription data and were prescribed at least one antidepressant primarily metabolized by CYP2D6 (fluoxetine, paroxetine, mirtazapine, or venlafaxine) and constituted the analytic cohort for drug-specific analyses. The mean age at index was 51.81 years (SD = 9.79), and 67.5% of participants were female (total *N* = 26,957). Within the analytic cohort, CYP2D6 normal metabolizers constituted the largest group (n = 13,585, 50.40%), followed by intermediate metabolizers (n = 11,044, 40.97%), poor metabolizers (n = 1965, 7.29%), and ultrarapid metabolizers (n = 363, 1.35%). Among antidepressants predominantly metabolized by CYP2D6 (fluoxetine, paroxetine, mirtazapine, and venlafaxine), fluoxetine was the most frequently prescribed (n = 15,572, 57.77%), followed by paroxetine (n = 5718, 21.21%), mirtazapine (n = 3340, 12.39%), and venlafaxine (n = 2327, 8.63%). The distribution of metabolizer profiles and CYP2D6-metabolized drugs prescription, as well as basic demographic information, are presented in Table 1. CYP2D6 metabolizer phenotype distributions were comparable across drug-specific cohorts (χ^2^(9) = 10.50, *p* = 0.31), indicating that phenotype composition did not differ systematically by antidepressant prescribed.

### 2.2. Associations Between Metabolizer Profile and Antidepressant Discontinuation

**Figure 1:**
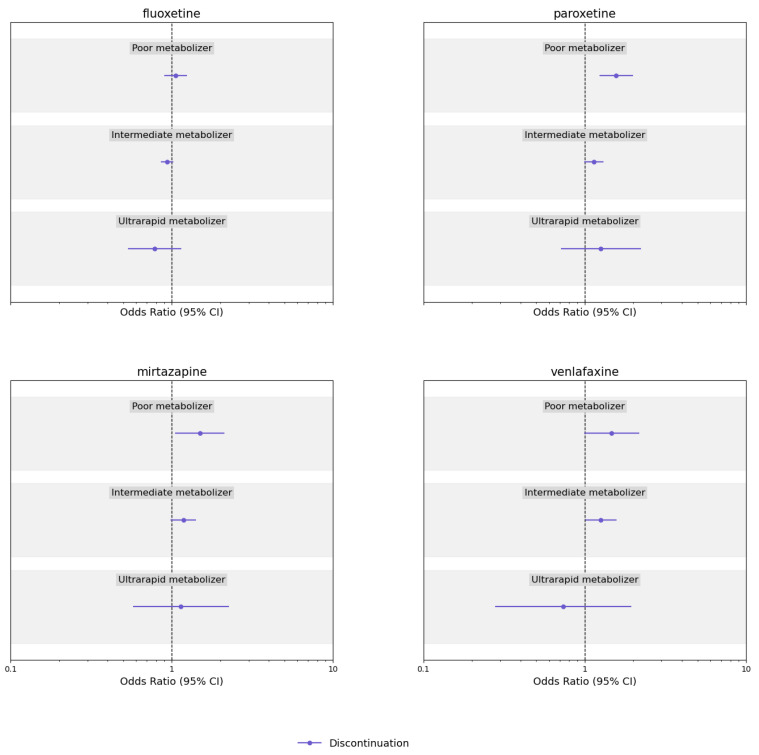
Association between CYP2D6 metabolizer phenotype and antidepressant discontinuation across CYP2D6-metabolized antidepressants. Forest plots show odds ratios (ORs) and 95% confidence intervals (CIs) for early treatment discontinuation comparing CYP2D6 poor, intermediate, and ultrarapid metabolizers with normal metabolizers (reference group), stratified by antidepressant (fluoxetine, paroxetine, mirtazapine, and venlafaxine). Estimates were derived from logistic regression models adjusted for age at index prescription, sex, and Townsend deprivation index. The vertical dashed line indicates the null effect (OR = 1).

Subsequently, the association between CYP2D6 metabolizer status and antidepressant discontinuation was investigated (Figure 1). Among individuals prescribed paroxetine, poor metabolizers were significantly more likely to discontinue treatment compared with normal metabolizers (OR = 1.57, 95% CI = 1.24–1.99, *p* < 0.001), corresponding to discontinuation rates of 24.0% in normal metabolizers and 31.0% in poor metabolizers. For venlafaxine, phenotype-specific models indicated increased discontinuation among reduced metabolizers, including intermediate versus normal metabolizers (OR = 1.26, 95% CI = 1.01–1.57, *p* = 0.043), corresponding to discontinuation rates of 21.5% and 24.7%, respectively. However, this finding should be interpreted with caution given the modest effect size and the absence of formal correction for multiple testing. For mirtazapine, poor metabolizers were more likely to discontinue treatment compared with normal metabolizers (OR = 1.49, 95% CI = 1.05–2.12, *p* = 0.026), corresponding to discontinuation rates of 22.6% in normal metabolizers and 26.8% in poor metabolizers. As with venlafaxine, this association is based on a nominal *p*-value and modest effect size and should be interpreted cautiously. No significant associations between CYP2D6 metabolizer status and antidepressant discontinuation were observed for fluoxetine. Full model results are provided in Supplementary Table S1.

### 2.3. Discontinuation Timing Analyses

#### 2.3.1. Immediate Discontinuation

Because paroxetine, mirtazapine, and venlafaxine showed the clearest discontinuation associations in the main analysis, we next examined whether CYP2D6 effects were concentrated at treatment initiation. In immediate discontinuation models adjusted for age, sex, and Townsend deprivation index, CYP2D6 metabolizer status was associated with immediate discontinuation for paroxetine and venlafaxine. Among paroxetine users, intermediate metabolizers had 16% higher odds of immediate discontinuation compared with normal metabolizers (OR 1.16, 95% CI 1.02–1.33; *p* = 0.029), and poor metabolizers had 63% higher odds (OR 1.63, 95% CI 1.29–2.05; *p* < 0.001), whereas ultrarapid metabolizers showed no significant association (*p* = 0.33). For venlafaxine, poor metabolizers were also more likely to discontinue immediately (OR 1.56, 95% CI 1.06–2.31; *p* = 0.025), while intermediate and ultrarapid metabolizers were not significantly different from normal metabolizers (*p* = 0.18 and *p* = 0.41, respectively). For mirtazapine, immediate discontinuation estimates were directionally elevated for poor metabolizers but did not reach statistical significance (poor vs. normal OR 1.35, 95% CI 0.96–1.89; *p* = 0.085). Full model estimates are provided in Supplementary Table S2.

#### 2.3.2. Immediate vs. Late Among Discontinuers

Among individuals who discontinued each medication, CYP2D6 status influenced the timing of discontinuation for paroxetine. Poor metabolizers were more likely to discontinue immediately rather than later compared with normal metabolizers (OR 2.12, 95% CI 1.15–3.89; *p* = 0.015), while intermediate metabolizers showed a similar but non-significant trend (OR 1.31, 95% CI 0.98–1.77; *p* = 0.073). No significant differences in immediate versus late discontinuation were observed for venlafaxine or mirtazapine (all *p* > 0.14), noting that confidence intervals were wide for several contrasts. Complete regression results are shown in Supplementary Table S3.

#### 2.3.3. Cox Maintenance-Phase Model

In time-to-discontinuation analyses excluding immediate discontinuation events (maintenance phase), CYP2D6 metabolizer status was not associated with treatment persistence for paroxetine, venlafaxine, or mirtazapine (all *p* > 0.05). For venlafaxine, the intermediate versus normal contrast showed a borderline trend toward shorter persistence (HR 1.53, 95% CI 0.97–2.41; *p* = 0.066), while other contrasts were not significant. These findings suggest that CYP2D6-related differences in discontinuation for these drugs are primarily concentrated in the early treatment period, particularly at treatment initiation, rather than during maintenance-phase persistence. Full Cox model estimates are provided in Supplementary Table S4.

### 2.4. Switching

**Figure 2:**
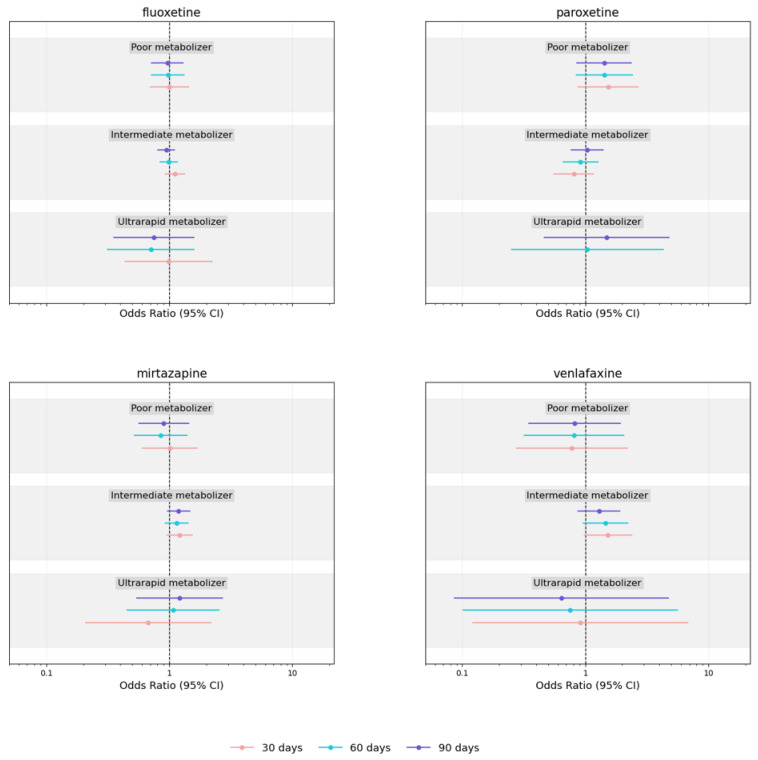
Association between CYP2D6 metabolizer phenotype and antidepressant switching across CYP2D6-metabolized antidepressants. Forest plots display odds ratios (ORs) and 95% confidence intervals (CIs) for switching to a different antidepressant among CYP2D6 poor, intermediate, and ultrarapid metabolizers compared with normal metabolizers (reference group), stratified by antidepressant (fluoxetine, paroxetine, mirtazapine, and venlafaxine). Switching was assessed within predefined follow-up windows of 30, 60, and 90 days following the index prescription. Estimates were derived from logistic regression models adjusted for age at index prescription, sex, and Townsend deprivation index. The vertical dashed line indicates the null effect (OR = 1).

No statistically significant associations were observed between CYP2D6 metabolizer phenotype and switching outcomes (Figure 2). However, among individuals prescribed venlafaxine, intermediate metabolizers showed a nominally increased likelihood of switching within 30 and 60 days compared with normal metabolizers (30-day switching: OR = 1.53, 95% CI = 0.97–2.42; *p* = 0.068; 60-day switching: OR = 1.46, 95% CI = 0.95–2.23; *p* = 0.081), although these associations did not reach conventional significance thresholds. Full model results are provided in Supplementary Table S5.

### 2.5. Side Effects

**Figure 3:**
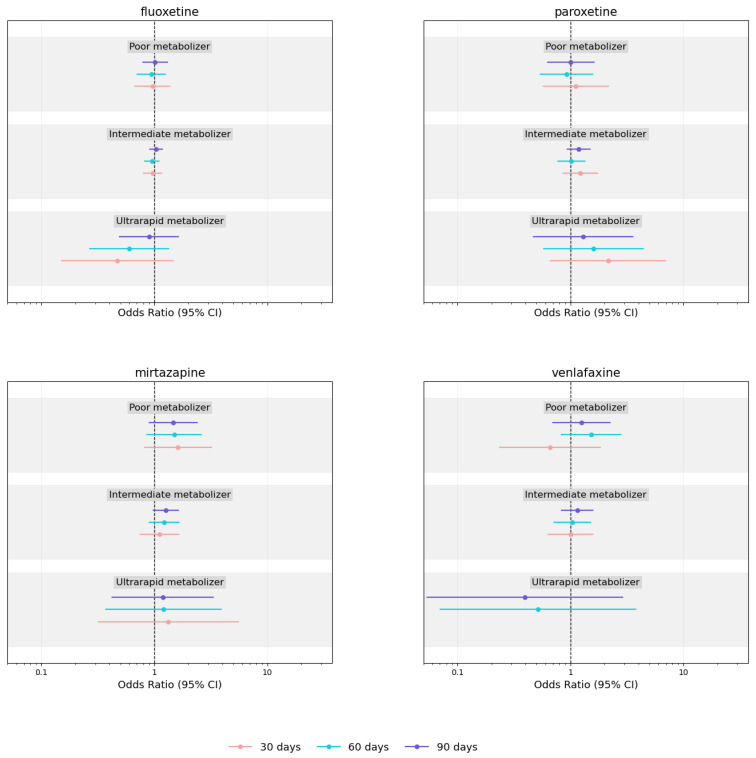
Association between CYP2D6 metabolizer phenotype and antidepressant side effects across CYP2D6-metabolized antidepressants. Forest plots display odds ratios (ORs) and 95% confidence intervals (CIs) for the occurrence of reported side effects among CYP2D6 poor, intermediate, and ultrarapid metabolizers compared with normal metabolizers (reference group), stratified by antidepressant (fluoxetine, paroxetine, venlafaxine, and mirtazapine). Side effects were assessed within predefined follow-up windows of 30, 60, and 90 days following the index prescription and were derived from UKU-related adverse event codes in primary care records, conditional on the absence of baseline events. Estimates were obtained from logistic regression models adjusted for age at index prescription, sex, and Townsend deprivation index. The vertical dashed line indicates the null effect (OR = 1).

Subsequently, the associations between CYP2D6 metabolizer phenotype and antidepressant side effects within 30-, 60-, and 90-day windows were examined (UKU-coded clinical events after treatment initiation, excluding events recorded in the 30 days before initiation; Figure 3), adjusting for age, sex, and Townsend. Overall, no significant associations were observed between CYP2D6 metabolizer status and side effects for fluoxetine, paroxetine, or venlafaxine across any time window (all *p* > 0.05). For mirtazapine, point estimates suggested higher odds of side effects among poor metabolizers compared with normal metabolizers across 30–90 days (e.g., OR 1.61 at 30 days, OR 1.50 at 60 days, OR 1.47 at 90 days), but these did not reach statistical significance (all *p* > 0.05); the strongest trend was observed for intermediate metabolizers at 90 days (OR 1.26, 95% CI 0.97–1.65, *p* = 0.089). Full model estimates are provided in Supplementary Table S6.

### 2.6. Sensitivity Analyses

To evaluate whether prescribing patterns influenced side effect findings, models were additionally adjusted for maximum prescribed dose and dose escalation. For venlafaxine, higher maximum dose was associated with increased, but stable side effects across all follow-up windows. Venlafaxine dose escalation (maximum dose > starting dose) was likewise associated with increased side effects at all time points. For mirtazapine, higher maximum dose was associated with increased side effects across all windows. Dose escalation was associated with increased side effects at 60 days, with similar but non-significant estimates at 30 days. For fluoxetine, maximum dose was not associated with side effects in any time window. Dose escalation was not associated with side effects at 30 or 60 days but was associated with increased side effects only at 90 days. Dose-related associations were not consistently observed for paroxetine. Importantly, inclusion of maximum dose did not materially alter the direction or significance of CYP2D6 phenotype associations, supporting robustness to dose adjustment (Supplementary Tables S7–S9).

To assess specificity, negative control analyses in antidepressants not primarily metabolized by CYP2D6 (citalopram, escitalopram, and sertraline) showed no association between CYP2D6 phenotype and early treatment change (Supplementary Table S10). Additionally, inclusion of CYP2D6-by-sex interaction terms in immediate discontinuation models provided no consistent evidence of effect modification by sex.

In ancestry sensitivity analyses including the full cohort irrespective of genetic ancestry, effect estimates were largely consistent with the primary analysis. The association between CYP2D6 poor metabolizer status and paroxetine discontinuation remained significant and directionally similar. Across venlafaxine and mirtazapine, point estimates were comparable, with some contrasts shifting around nominal significance thresholds (Supplementary Table S11). In analyses restricted to participants with depressive disorders, venlafaxine discontinuation associations were directionally consistent and strengthened. For paroxetine and mirtazapine, effect estimates remained directionally similar but were less precise, consistent with reduced sample size. Ultrarapid metabolizer contrasts were sparse in the restricted cohort and yielded unstable estimates, these were therefore not emphasized in sensitivity reporting (Supplementary Table S12).

## 3. Discussion

In this large population-based study, longitudinal prescription data from the UK Biobank were assessed for the relationship between CYP2D6 metabolizer status and antidepressant treatment discontinuation. Across several complementary outcome definitions, it was observed that reduced CYP2D6 metabolic capacity was associated with early antidepressant discontinuation for paroxetine, venlafaxine, and mirtazapine. In contrast, CYP2D6 phenotype was not associated with maintenance-phase persistence once treatment had been established. Taken together, these findings suggest that CYP2D6 variation may primarily influence very early treatment tolerability or treatment adaptation rather than longer-term continuation. This overall pattern is broadly consistent with current pharmacogenetic guidelines that recognize the role of CYP2D6 variation in shaping antidepressant exposure and dosing considerations at treatment initiation, particularly for paroxetine and venlafaxine [8,19,20]. At the same time, the observed association for mirtazapine extends the available evidence for antidepressants where formal CYP2D6-guided prescribing recommendations remain limited, while the absence of association for fluoxetine aligns with the lack of current pharmacogenetic guidance for this drug [8,20].

The strongest and most consistent associations were observed for paroxetine. Poor metabolizers exhibited increased odds of early discontinuation and were more likely to discontinue immediately rather than later compared with normal metabolizers. Beyond the primary discontinuation models, timing analyses indicated that CYP2D6 effects were concentrated at treatment initiation. In contrast, when immediate discontinuation events were excluded and treatment persistence was evaluated during the maintenance phase using time-to-event modeling, CYP2D6 metabolizer status was not associated with time to discontinuation. Together, these findings support a pattern in which reduced CYP2D6 metabolic capacity primarily contributes to very early treatment discontinuation rather than longer-term persistence once treatment is established. This interpretation is biologically plausible. Paroxetine is extensively metabolized by CYP2D6 and is also a potent CYP2D6 inhibitor, such that reduced enzymatic activity may lead to elevated plasma concentrations and reduced tolerability [21,22]. Increased exposure in poor metabolizers may therefore increase the likelihood of early adverse effects or subjective intolerance, resulting in treatment discontinuation shortly after initiation. This aligns with existing pharmacogenetic guidance for paroxetine, although recommendations differ somewhat between CPIC and the DPWG. CPIC recommends dose reduction and slower titration for CYP2D6 poor metabolizers, and consideration of an alternative antidepressant for ultrarapid metabolizers, whereas the DPWG recommends avoiding paroxetine in ultrarapid metabolizers but does not currently recommend action for poor or intermediate metabolizers [19,23]. In this context, our findings provide population-based observational evidence supporting the clinical relevance of CYP2D6 poor metabolizer status for early paroxetine discontinuation. However, the number of ultrarapid metabolizers in our cohort was relatively small, limiting our ability to draw firm conclusions regarding this phenotype group.

Venlafaxine also demonstrated increased early discontinuation among reduced metabolizers, particularly in analyses focusing on immediate discontinuation. Although effect sizes were more modest compared with paroxetine, the directionally consistent findings and replication in the depression-restricted cohort are suggestive; these results should nonetheless be interpreted with caution given the nominal *p*-values and absence of formal multiple testing correction. This interpretation is also broadly consistent with recent DPWG and CPIC recommendations, which advise avoiding venlafaxine in CYP2D6 poor and intermediate metabolizers and considering an alternative antidepressant less dependent on CYP2D6 metabolism [8,20].

For mirtazapine, poor metabolizers showed increased odds of early discontinuation, although associations were less consistent across timing analyses. Mirtazapine is metabolized by multiple CYP enzymes, including CYP2D6, but also CYP1A2, and CYP3A4. The observed associations may therefore reflect a partial contribution of CYP2D6-dependent metabolism within a broader metabolic network rather than exclusive pathway dependence. Current pharmacogenetic guidelines acknowledge a CYP2D6-mirtazapine gene–drug interaction but do not recommend therapy adjustment, largely due to inconsistent evidence regarding clinical consequences [20]. While several pharmacokinetic studies have reported altered plasma concentrations across CYP2D6 metabolizer groups, prior clinical studies have been small and have produced mixed findings regarding treatment effectiveness and adverse effects [24,25,26]. In this context, our findings provide population-based observational evidence suggesting that CYP2D6 poor metabolizer status may contribute to early treatment discontinuation for mirtazapine, although the modest and less consistent effect sizes, combined with nominal *p*-values and the absence of multiple testing correction, indicate that these findings require replication before conclusions can be drawn about the clinical relevance of CYP2D6 for mirtazapine. In contrast, no significant associations were observed for fluoxetine. This absence of association may reflect the drug’s long half-life, active metabolite, and complex pharmacokinetic profile [27]. In addition, fluoxetine is a potent inhibitor of CYP2D6, which may attenuate genotype-related differences in enzyme activity during treatment and thereby reduce the observable impact of CYP2D6 variability on early treatment persistence [19]. Consistent with this observation, current pharmacogenetic guidelines from both CPIC and the DPWG do not provide specific CYP2D6-guided prescribing recommendations for fluoxetine [8,19].

Consistent with the early-discontinuation pattern observed across drugs, CYP2D6 phenotype was not associated with maintenance-phase time-to-discontinuation. This suggests that the influence of metabolic variation is concentrated during the initiation phase, potentially mediated by early pharmacokinetic exposure differences rather than sustained differences in long-term treatment response.

Treatment switching was assessed as an alternative indicator of early treatment modification. In contrast to discontinuation, CYP2D6 metabolizer status was not consistently associated with switching between antidepressants. This suggests that the pharmacogenetic effects observed in this study may primarily manifest as early treatment cessation rather than structured switching to an alternative antidepressant. One possible explanation is that switching events are more strongly influenced by clinician decision-making, treatment guidelines, or longer-term assessment of treatment response, whereas early discontinuation may more directly reflect acute tolerability or early subjective treatment experiences.

We did not observe robust associations between CYP2D6 phenotype and recorded side effects as captured in available UKU records. While dose-related variables were associated with side effect reporting for venlafaxine and mirtazapine, adjustment for dose did not materially alter metabolizer-discontinuation associations. This pattern may indicate that early discontinuation reflects factors not fully captured by coded adverse event data, including subjective tolerability, early perceptions of inefficacy, or clinician-driven treatment modification. Additionally, UKU assessments are not systematically recorded for every patient in routine clinical practice. As a result, the absence of a recorded UKU event cannot be interpreted as the absence of side effects. This likely leads to under-ascertainment of adverse events and reduces sensitivity to detect pharmacogenetic associations with tolerability, potentially biasing effect estimates toward the null. Sensitivity analyses demonstrated consistent findings across ancestry groups and in a depression-restricted cohort, supporting the robustness of the primary associations. Negative control analyses in antidepressants not primarily metabolized by CYP2D6 showed no associations, reinforcing the drug-specific nature of the observed findings.

Importantly, these results should be interpreted as supportive observational evidence rather than direct evidence for clinical implementation. Prescription-based outcomes capture real-world prescribing behavior but cannot fully disentangle pharmacological intolerance, patient preference, or clinician decision-making.

Several additional methodological considerations should be noted. Immediate discontinuation was defined as cessation on the day of the index prescription and interpreted as reflecting failure to continue beyond the initial prescription. While this pattern may reflect early intolerance or rapid treatment modification, it may also capture individuals who never initiated treatment or administrative artifacts in prescription records. Nevertheless, the drug-specific pattern observed for paroxetine and the consistency across analyses support a pharmacogenetic contribution.

A further consideration relates to multiple testing. We did not formally adjust for multiple comparisons across antidepressants, outcomes, and time windows. Given the exploratory nature of evaluating several related prescription-based endpoints and the absence of clear consensus regarding the most appropriate correction strategy in this context, particularly when outcomes reflect overlapping dimensions of treatment persistence, we elected to report unadjusted results [28,29]. While this approach increases the possibility of false-positive findings, the consistency, biological plausibility, and drug-specific nature of the paroxetine associations strengthen confidence in a true pharmacogenetic signal. Nevertheless, replication in independent cohorts will be important.

This study has several notable strengths. First, the analyses were conducted in a large population-based cohort with longitudinal prescription records from the UK Biobank, providing substantial statistical power to evaluate pharmacogenetic associations in a real-world clinical context. Second, we implemented a structured outcome framework based on multiple complementary prescription-derived endpoints, including discontinuation, switching, and timing analyses, which allowed us to localize pharmacogenetic effects to the treatment initiation phase. Third, the observed associations were drug-specific and biologically consistent with the pharmacology of CYP2D6-mediated metabolism, strengthening the plausibility of the findings. At the same time, several limitations should be acknowledged. UK Biobank participants are not fully representative of the general UK population and tend to be healthier and socioeconomically advantaged, which may limit generalizability [30]. Furthermore, the cohort may include individuals with bipolar disorder in a depressive phase, as diagnostic ascertainment relied on ICD-10 coded records without structured clinical interviews; future studies with more granular diagnostic data could examine whether CYP2D6-metabolizer phenotype associations with discontinuation differ by specific diagnostic subtype. In addition, prescription records cannot capture whether dispensed medications were taken by patients or the clinical reasoning underlying treatment discontinuation or modification. A limitation of this study is the restricted set of covariates included in the primary models. Although we adjusted for age, sex, and socioeconomic status, we were unable to fully account for clinically relevant factors such as baseline disease severity, psychiatric comorbidities, prior antidepressant exposure, concomitant medications, and prescribing context, which may influence discontinuation patterns. Residual confounding by unmeasured clinical factors cannot be excluded, and side effects are likely under-recorded in routine healthcare data. Finally, prescription-based outcomes represent proxies for treatment response and tolerability rather than direct clinical measures. Additionally, metabolizer phenotypes were inferred from genotype data rather than empirically measured via phenotyping probes, and may therefore not fully capture interindividual variability in enzyme activity arising from non-genetic sources such as drug–drug interactions or environmental influences.

Future implementation of CYP2D6-guided prescribing could be complemented by therapeutic drug monitoring (TDM), which measures plasma drug concentrations and can capture phenoconversion due to drug–drug interactions that genotyping alone cannot detect. For paroxetine, venlafaxine, and mirtazapine, established plasma concentration reference ranges exist, supporting the feasibility of TDM-guided dose individualization in clinical practice. Future studies incorporating biomarkers of adverse effects alongside genotype data, and combining CYP2D6 genotyping with phenotypic assessments, could further elucidate the mechanisms underlying CYP2D6-related discontinuation and improve prediction of drug response.

In summary, our findings suggest that reduced CYP2D6 metabolic capacity is associated with an increased likelihood of early antidepressant discontinuation, particularly for paroxetine, with additional signals observed for venlafaxine and mirtazapine. These results support a model in which CYP2D6 genetic variation exerts its strongest clinical influence during treatment initiation rather than during long-term maintenance. While effect sizes were modest, they were consistent with pharmacological expectations and observed in a large real-world cohort. Together, these findings provide population-based support for the clinical relevance of CYP2D6 variation in antidepressant prescribing while underscoring the need for further studies with harmonized outcome definitions and prospective designs to clarify the clinical utility of genotype-guided treatment strategies.

## 4. Data & Methods

### 4.1. Study Population

Study data were obtained from the UK Biobank, a large prospective population-based cohort that recruited approximately 500,000 participants aged 40 to 69 years from across the United Kingdom between 2006 and 2010 [31]. For the present analyses, we focused on participants with available genetic data and linked primary care prescription records for antidepressant medications. Diagnostic information was ascertained from linked primary care and hospital inpatient records using ICD-10 codes; no formal structured clinical interviews or DSM-based assessments were conducted as part of the UK Biobank protocol. Antidepressant exposure episodes were defined using primary care prescription records for fluoxetine (ATC N06AB03), paroxetine (ATC N06AB05), venlafaxine (ATC N06AX16), and mirtazapine (ATC N06AX11). For each antidepressant, the first recorded prescription was treated as the index prescription. This approach enabled standardized identification of treatment initiation across participants while minimizing bias related to prior treatment history. Participants were included in drug-specific analyses if they had sufficient prescription data to define treatment initiation and subsequent follow-up outcomes within predefined time windows. Individuals were excluded from specific analyses if required genetic, outcome, or covariate data were missing for the corresponding model, ensuring consistency across regression analyses while maximizing the available sample size for each antidepressant examined.

### 4.2. CYP2D6 Genotyping and Metabolizer Phenotype

CYP2D6 genotypes and metabolizer phenotypes were derived from UK Biobank pharmacogenetic data generated using the DRAGEN pharmacogene caller pipeline implemented centrally by UK Biobank. DNA samples were extracted from whole blood and genotyped using either the Affymetrix UK Biobank Axiom or UK BiLEVE Axiom arrays, followed by imputation and subsequent pharmacogene calling [32]. The DRAGEN CYP2D6 caller assigns star allele haplotypes and corresponding diplotypes for each participant based on variant information across the CYP2D6 locus. For the present analyses, we used the star allele diplotypes provided by UK Biobank and translated them into predicted metabolizer phenotypes according to the guidelines of the Dutch Pharmacogenetics Working Group (DPWG). DPWG guidelines classify CYP2D6 metabolic capacity based on functional allele combinations [19,20]. Participants were categorized into four metabolizer groups: poor metabolizers, intermediate metabolizers, normal metabolizers, and ultrarapid metabolizers. Normal metabolizers served as the reference group in all analyses. Individuals whose CYP2D6 star allele assignments were missing, indeterminate, or could not be confidently mapped to a DPWG-defined phenotype were excluded from CYP2D6-specific analyses to minimize phenotype misclassification. It should be noted that the metabolizer phenotypes used throughout this study represent genotype-predicted classifications inferred from diplotype data, and do not reflect empirically measured metabolic capacity. This approach is consistent with standard CPIC and DPWG nomenclature, in which genotype-derived phenotype assignments form the basis for clinical recommendations.

### 4.3. Antidepressant Switching and Discontinuation Outcomes

Antidepressant switching and discontinuation outcomes were derived from longitudinal primary care prescription records, which provide detailed information on medication initiation, continuation, and changes over time. Antidepressant discontinuation was defined as treatment cessation following a brief exposure period. Two alternative definitions were explored based on prescription duration and refill patterns, capturing both failure to continue treatment beyond the initial prescription, and an abrupt stoppage of treatment following a prescription period of less than 8 weeks. Accordingly, a single binary outcome was used for primary analyses, indicating discontinuation by either definition (failure to refill beyond initial prescription or cessation before 8 weeks) [17]. Switching was defined as the initiation of a different antidepressant following the index prescription within predefined time windows of 30, 60, or 90 days. This operationalization captured any different antidepressant prescription occurring within the specified window, regardless of whether treatment overlap was present, thereby reflecting real-world prescribing patterns in which cross-titration or temporary combination therapy may occur. Primary analyses therefore focused on this switching definition in order to maximize sensitivity to clinically relevant treatment changes.

Given that significant associations with early discontinuation were observed for paroxetine, mirtazapine, and venlafaxine, we performed additional exploratory drug-specific analyses to characterize discontinuation timing. These included analyses of immediate discontinuation, immediate-versus-late discontinuation among discontinuers, and time-to-discontinuation during the maintenance phase. For these drug-specific analyses, immediate discontinuation was defined as failure to continue treatment beyond the initial prescription. Late discontinuation was defined as treatment cessation occurring after at least one day of continued treatment. Among discontinuers of each drug, we modelled immediate versus late discontinuation. For time-to-discontinuation analyses, immediate discontinuation events were excluded, and time was defined as days from the index prescription to discontinuation.

### 4.4. Side Effects Outcome

Side effect outcomes were derived by linking antidepressant prescription records to longitudinal clinical event data annotated with UKU-related adverse effect codes [33]. For each index prescription, we identified whether any UKU-flagged clinical event was recorded after treatment initiation within predefined follow-up windows of 30, 60, or 90 days. This approach was designed to capture early adverse effects that are most likely to influence treatment persistence and patient tolerability during the initial treatment phase. To reduce misclassification due to pre-existing symptoms, ongoing adverse events, or conditions unrelated to antidepressant exposure, a baseline period was defined as the 30 days prior to treatment initiation. Side effect outcomes were only considered incident events if no UKU-flagged clinical event was recorded during this baseline period. Primary side effect outcomes were defined as binary indicators reflecting the occurrence of at least one UKU-flagged event following treatment initiation within each time window. Participants with no linked UKU-flagged clinical events during follow-up were coded as having no reported side effects. Analyses were conducted separately for each antidepressant.

### 4.5. Statistical Analyses

Associations between CYP2D6 metabolizer status and antidepressant treatment outcomes were assessed using logistic regression models, stratified by antidepressant. CYP2D6 metabolizer phenotype was modelled as a four-category variable comprising poor, intermediate, normal, and ultrarapid metabolizers, with normal metabolizers serving as the reference group in all analyses. All models were adjusted for sex, age at index prescription, and Townsend deprivation index at recruitment. To evaluate maintenance-phase persistence beyond the initial prescription, time-to-discontinuation was additionally analyzed using Cox proportional hazards regression. Logistic regression results are reported as odds ratios (ORs), and Cox regression results as hazard ratios (HRs), each with corresponding 95% confidence intervals (CIs). Statistical analyses were conducted using Python (version 3.12.2) (statsmodels), with model diagnostics and convergence checks performed for all primary analyses. Full model outputs, including coefficient estimates, confidence intervals, and *p*-values for all covariates, are provided in the Supplementary Materials.

### 4.6. Sensitivity Analyses

Several sensitivity analyses were conducted to assess the robustness and specificity of the primary findings. First, to evaluate whether dose-related prescribing patterns influenced associations with side effects, models were additionally adjusted for maximum prescribed dose and dose escalation following treatment initiation, alongside the demographic covariates included in the primary analyses. Maximum dose was defined as the highest recorded dose during follow-up, and dose escalation as any increase relative to the initial prescription. These variables were included to account for potential confounding arising from clinician-driven dose adjustments in response to perceived efficacy or tolerability. Second, to assess specificity of the discontinuation findings, negative control analyses were performed using antidepressants not primarily metabolized by CYP2D6 (citalopram, escitalopram, and sertraline), applying an analogous early treatment change outcome. We also evaluated potential effect modification by sex by including CYP2D6-by-sex interaction terms in immediate discontinuation models. Finally, to examine the impact of cohort definition, we conducted ancestry and diagnosis sensitivity analyses. Primary analyses were restricted to participants of genetically defined European ancestry to reduce population heterogeneity in pharmacogene calling and allele frequency distributions. To assess generalizability, we repeated the primary drug-stratified discontinuation and switching models in the full cohort irrespective of ancestry. To evaluate potential confounding by treatment indication, we repeated the primary models in a cohort restricted to participants with depressive disorders (ICD-10 codes F32, F33, and F34).

## 5. Conclusions

In this large population-based study using UK Biobank data, we demonstrate that CYP2D6 metabolizer phenotype is associated with early antidepressant treatment discontinuation for three of the four CYP2D6-metabolized antidepressants examined: paroxetine, venlafaxine, and mirtazapine, with the strongest associations observed for paroxetine. Timing analyses suggested that these effects were concentrated at treatment initiation, with no evidence of CYP2D6 association with maintenance-phase persistence after excluding immediate discontinuation. Poor metabolizers showed a higher likelihood of early discontinuation at treatment initiation compared with normal metabolizers. In contrast, CYP2D6 metabolizer status was not associated with maintenance-phase treatment persistence, and associations with antidepressant switching were limited. Associations with recorded side effects were also limited and should be interpreted cautiously given incomplete recording of UKU assessments in routine clinical practice. Together, these findings suggest that CYP2D6 metabolizer status may primarily influence tolerability-driven treatment failure at initiation rather than downstream switching decisions, highlighting early discontinuation as a potentially informative proxy of pharmacokinetically driven treatment response in prescription-based datasets. Our results provide population-based support for the clinical relevance of CYP2D6 variation in antidepressant prescribing, particularly for CYP2D6-dependent antidepressants such as paroxetine, venlafaxine, and mirtazapine.

## Figures and Tables

**Table 1:** Distribution of CYP2D6 metabolizer phenotypes, antidepressant prescriptions, and demographic characteristics in the UK Biobank analytic cohort. The table presents the number and percentage of participants in each CYP2D6 metabolizer category (normal, intermediate, poor, and ultrarapid) stratified by antidepressant prescribed (fluoxetine, paroxetine, mirtazapine, and venlafaxine), as well as in the total analytic sample. The total number of individuals prescribed each CYP2D6-metabolized antidepressant is shown, together with basic demographic characteristics, including age at index prescription (mean ± standard deviation) and sex distribution. The index prescription was defined as the first qualifying prescription for each antidepressant. Percentages are calculated within each drug-specific cohort unless otherwise indicated. Chi-square test of independence revealed no significant difference in CYP2D6 metabolizer phenotype distribution across antidepressant cohorts (χ^2^(9) = 10.50, *p* = 0.31), indicating comparable pharmacogenetic composition across drug-specific analytic samples.

	Fluoxetine	Paroxetine	Mirtazapine	Venlafaxine	Total Sample
Metabolizer Phenotype					
Normal, *N* (%)	7792 (50.04%)	2898 (50.68%)	1717 (51.41%)	1178 (50.62%)	13,585 (50.40%)
Intermediate, *N* (%)	6400 (41.10%)	2333 (40.80%)	1366 (40.90%)	945 (40.61%)	11,044 (40.97%)
Poor, *N* (%)	1170 (7.51%)	420 (7.35%)	207 (6.20%)	168 (7.22%)	1965 (7.29%)
Ultrarapid, *N* (%)	210 (1.35%)	67 (1.17%)	50 (1.50%)	36 (1.55%)	363 (1.35%)
*N*	15,572	5718	3340	2327	26,957
Demographics					
Age at index (mean ± SD)	51.14 ± 9.38	48.42 ± 8.51	60.02 ± 9.70	52.87 ± 8.94	51.81 ± 9.79
Sex (% female)	69.74%	67.72%	56.26%	67.68%	67.5%

## Data Availability

The original contributions presented in the study are included in the article/Supplementary Materials, further inquiries can be directed to the corresponding authors.

## Supplementary Materials

The following supporting information can be downloaded at: https://www.mdpi.com/article/10.3390/ph19071028/s1, the following supplementary tables are attached to this manuscript as a separate file: Table S1. Logistic regression models of CYP2D6 metabolizer phenotype and antidepressant discontinuation, stratified by drug, Table S2. Logistic regression models of CYP2D6 metabolizer phenotype and immediate antidepressant discontinuation at treatment initiation, Table S3. Logistic regression models of CYP2D6 metabolizer phenotype and immediate versus late discontinuation among discontinuers, Table S4. Cox proportional hazards models of time to antidepressant discontinuation during the maintenance phase, Table S5. Logistic regression models of CYP2D6 metabolizer phenotype and antidepressant switching within 30, 60, and 90 days, Table S6. Logistic regression models of CYP2D6 metabolizer phenotype and antidepressant-related side effects within 30, 60, and 90 days, Table S7. Sensitivity analysis: association between maximum prescribed dose and antidepressant side effects, Table S8. Sensitivity analysis: association between dose escalation and antidepressant side effects, Table S9. Sensitivity analysis: CYP2D6 metabolizer phenotype and antidepressant side effects adjusted for dose-related variables, Table S10. Negative control analyses: association between CYP2D6 metabolizer phenotype and early treatment change for non-CYP2D6-metabolized antidepressants, Table S11. Sensitivity analysis: associations between CYP2D6 metabolizer phenotype and antidepressant discontinuation in the full cohort (all ancestries), Table S12. Sensitivity analysis: associations between CYP2D6 metabolizer phenotype and antidepressant discontinuation in participants with depressive disorders.
